# Longitudinal Cohort Event Monitoring of MMR and DT-IPV Vaccination at 9 Years of Age in The Netherlands

**DOI:** 10.3390/ph18111635

**Published:** 2025-10-29

**Authors:** Monika Raethke, Jeroen Gorter, Rachel Kalf, Leontine van Balveren, Sanne Boetzkes, Rana Jajou, Florence van Hunsel

**Affiliations:** 1 Netherlands Pharmacovigilance Centre Lareb, Goudsbloemvallei 7, 5237 MH ’s-Hertogenbosch, The Netherlands; 2 Department of PharmacoTherapy—Epidemiology & Economics, Groningen Research Institute of Pharmacy (GRIP), University of Groningen, Broerstraat 5, 9712 CP Groningen, The Netherlands

**Keywords:** national immunisation programme (NIP), vaccination, MMR (measles, mumps, and rubella) vaccine, DT-IPV (diphtheria, tetanus, and poliomyelitis) vaccine, adverse event following immunisation (AEFI), cohort event monitoring (CEM)

## Abstract

**Background/Objectives**: The Dutch National Immunisation Programme (NIP) aims to protect children against severe infectious diseases. As with all vaccines, adverse events following immunisation (AEFIs) may occur with the use of vaccines in the NIP. Safety of the vaccines is monitored by The Netherlands Pharmacovigilance Centre Lareb. This study aimed to systematically document AEFIs following administration of the MMR and DT-IPV vaccines, which are given simultaneously at the age of 9 years. **Methods**: A cohort event monitoring (CEM) study was performed, employing a longitudinal cohort design. Parents or guardians of 9-year-olds receiving the MMR and DT-IPV vaccines completed questionnaires following vaccination to report the presence or absence of AEFI. **Results**: AEFIs were reported for more than 73% of children given an MMR and DT-IPV vaccination. The great majority of the reported reactions were non-serious and self-limiting and consistent with those listed in the official product information for the MMR and DT-IPV vaccines. Injection site reactions were significantly more frequent at the site of the DT-IPV vaccination than the MMR vaccination. AEFIs were mostly perceived as little or moderately burdensome. **Conclusions**: AEFIs very frequently occurred after MMR and DT-IPV vaccination. This study provides further insight into the timing and duration of AEFIs after MMR and DT-IPV vaccination. In addition, detailed insight into the adverse event profile of these vaccines is provided, which helps to set realistic expectations for children and their parents or caretakers who follow the NIP and helps health professionals in their communication regarding AEFIs.

## 1. Introduction

The Dutch National Immunisation Programme (NIP) has the primary objective to optimally protect children against severe infectious diseases [1]. A Dutch study performing a historical time-series analysis of mortality and vaccination coverage indicated that as vaccination coverage increased, the contribution of vaccine-preventable diseases to overall mortality substantially declined [2]. Van Wijhe et al. [3] assessed the population-level effectiveness of childhood vaccination programmes in The Netherlands against diphtheria, pertussis, and poliomyelitis using mortality data from 1903 to 2012. By modelling pre-vaccination trends and comparing them to observed outcomes, the researchers estimated both direct and indirect effects. Diphtheria and pertussis vaccinations demonstrated substantial indirect (herd) protection, averting 14.9% and 32.1% of mortality burden, respectively, through indirect effects. All programmes achieved near-complete effectiveness within ten birth cohorts [3]. Other studies have found similar results for additional vaccines; for instance, the first national vaccination campaign against meningococcal-C disease almost immediately led to a sharp decrease in the number of patients with meningococcal serogroup-C disease [4], with long-lasting herd protection from reduced carriage of virulent meningococci [5]. These findings underscore the broad public health impact of vaccination, particularly for communicable diseases with potential for herd immunity.

The vaccination schedule for the NIP begins in infancy, with the final routine vaccination administered at the age of 14 [1]. Programme performance, including disease incidence and vaccination coverage, is systematically monitored. A comprehensive evaluation of the immunisation schedule may support further optimisation of the programme, aiming to achieve maximal protective efficacy with the minimal number of doses required [6]. Such revisions of the schedule may warrant enhanced post-vaccination surveillance to monitor the safety profile of the administered vaccines. Historically, children in The Netherlands received the second dose of the MMR (measles, mumps, and rubella) vaccine at the age of 9. The vaccine currently used is M-M-RVAXPRO^®^. This vaccination moment has been rescheduled to approximately 3 years of age since 2025. Concurrently, a DT-IPV (diphtheria, tetanus, and poliomyelitis) vaccine—REVAXIS^®^—was also administered at age 9. As of 2025, this DT-IPV vaccination will be postponed to age 14 [7].

As with all vaccines, adverse events following immunisation (AEFIs) may occur with the use of vaccines in the NIP [8,9]. The safety of the vaccines is monitored continuously by passive surveillance through the Dutch spontaneous reporting system maintained by the Dutch Pharmacovigilance Centre Lareb [10]. In addition, monitoring studies can be performed. In the past, the National Institute for Public Health and the Environment (RIVM) conducted a safety study on the diphtheria, tetanus, and inactivated poliomyelitis and MMR vaccines in 9-year-olds in 2010 [11]. Among these children, 83% reported a local reaction at the DT-IPV injection site, and 33% at the MMR injection site. Local reactions occurred in 87% of the children within a week after vaccination, more often at the DT-IPV (83%) than at the MMR site (33%). Approximately 21% reported AEFIs in the second week, aligning more closely with the MMR vaccine, which is a live attenuated vaccine, unlike the inactivated DT-IPV vaccine [11].

Since 2022 Lareb has followed children who received a NIP vaccine in multiple longitudinal cohort studies. Part of this cohort was the vaccination moment at 9 years of age. We aimed to systematically document AEFIs in children following administration of the MMR and DT-IPV vaccines at this age and provide updated information on the study performed fifteen years ago [11]. Data are collected and analysed to address the following research questions:

- What types of AEFIs occur, and at what frequency?
- Are any serious AEFIs reported that require medical intervention?
- What is the time to onset (TTO) and time to recovery (TTR) of AEFIs?
- Are there identifiable risk factors associated with the occurrence of AEFIs?

## 2. Results

### 2.1. Intake Questionnaire and General Participant Characteristics

**Figure 1:**
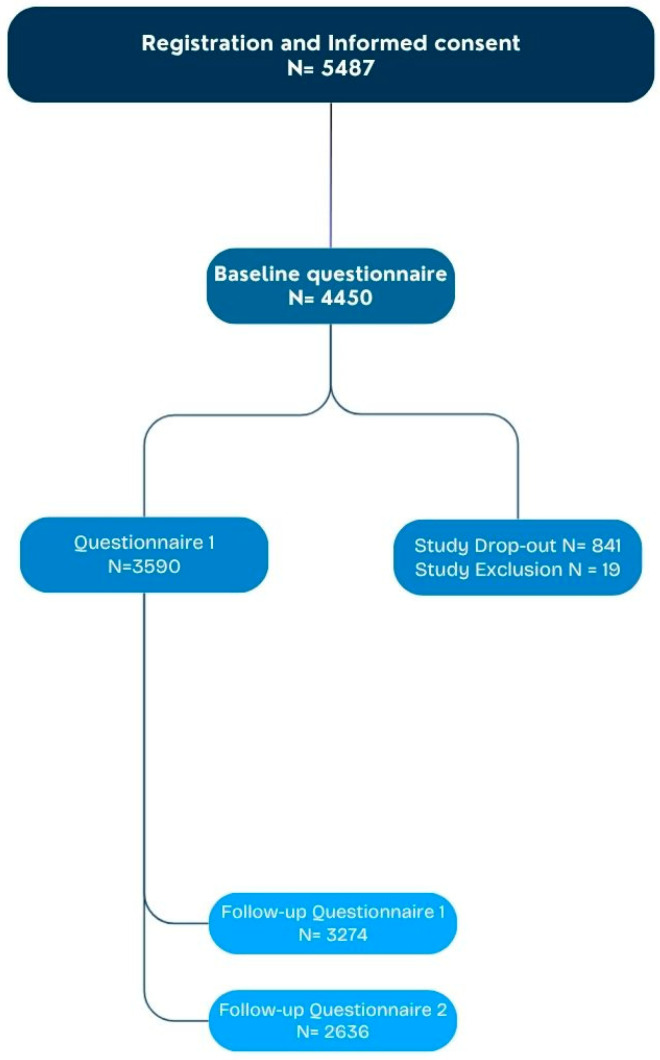
Flow-chart of participants in the study.

A total of 4450 participants completed the intake questionnaire for the MMR-DT-IPV vaccination (see Figure 1). Study attrition is based on participants dropping out of the study or being excluded due to not adhering to inclusion criteria. The first questionnaire on the occurrence of AEFIs was filled in by 3590 participants.

### 2.2. Characteristics of Participating Children and Their Household Situation

Table 1 presents the characteristics of the children for whom parents completed both the intake questionnaire and the first questionnaire. Several general characteristics are highlighted. The sex distribution within this cohort is nearly equal. Approximately 6.8% of the children for whom questionnaires were completed have at least one medical condition. The reported conditions are categorised by organ system and presented in Table 1.

**Figure 2:**
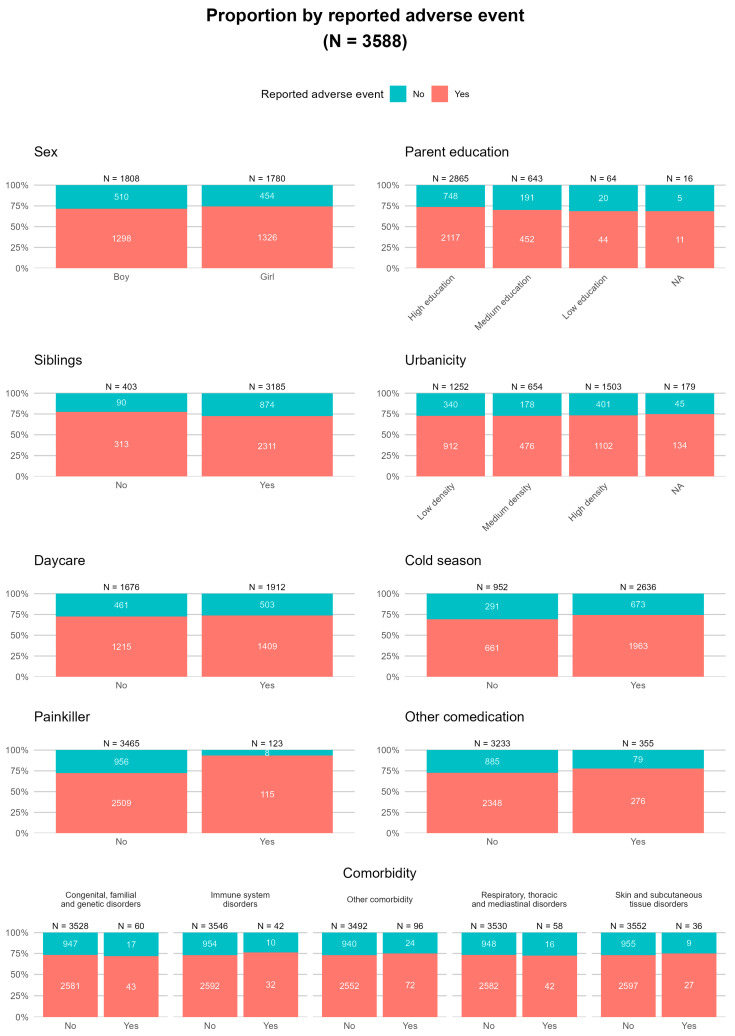
Characteristics of the children and their households, stratified by the occurrence of any AEFI or not (NA = Not Available). Other comorbidities were defined as conditions not relevant to the shown categories. The full list of existing medical conditions (MedDRA System Organ Class level) is shown in Table 1.

In Figure 2, the characteristics of the children and household characteristics of the families of participating children are presented, stratified by the proportion of children for whom at least one AEFI was reported. This figure excludes children for whom sex is unknown (n = 2). The majority of children come from households with at least one sibling (88.8%). The parents of participating children are generally highly educated: 79.8% have completed higher professional education or have a university-level degree. The households are spread throughout areas of low (34.9%), medium (18.2%), and high (41.9%) urbanicity in The Netherlands. Approximately half of the children attend after-school/day care (53.3%). Based on the vaccination date, 73.5% of the children were vaccinated in the fall and winter months (referred to as the ‘cold season’). For 123 children (3.4%), it was reported that they used some kind of pain relief around the vaccination. This could be prophylactic analgesics or for instance a cooling gel after the vaccination was given. For 355 children (9.9%) the use of concomitant medication was reported at the time of vaccination. This concomitant medication may have been used for either chronic treatment or short-term purposes, such as with antibiotics or antihistamines. Approximately 6.8% of the children in the cohort had at least one medical condition. It is possible to report multiple comorbidities per child. In Figure 2 we present the major comorbidity groups. In addition, parents were asked whether their child had recently received any other vaccinations. This was the case in a small number of children; the Hepatitis B vaccine was the most commonly given, with seven children receiving this vaccine (0.2%). Other vaccines given included the Hepatitis A vaccine and the Influenza vaccine, each administered five times (0.1%).

### 2.3. Reported Adverse Events Following Immunisation (AEFIs)

Table 2 presents the number of participants who reported one or more AEFI for their child. Among participants who completed at least one questionnaire following the baseline, 2625 (73.1%) reported that their child experienced at least one AEFI. In total, 5168 AEFIs were reported by 2625 participants.

The majority of predefined reported AEFIs consisted of local reactions at the injection site, such as pain or swelling (52.7%). Additionally, AEFIs such as headache (18.0%) and pyrexia (13.8%) were frequently reported.

In addition to the predefined AEFIs in the questionnaire, other AEFIs were reported, including fatigue (4.6%), muscle pain (4.5%), and abdominal pain (2.9%). An overview of the predefined AEFIs and the top 10 most frequently reported non-predefined AEFIs is provided in Table 2.

In Table 3 the number of children is given with one or more injection site reactions. In Table 3 these reactions were stratified to the left or right side according to the vaccination guidelines for the DT-IPV and MMR vaccines. The proportion of injection site reactions occurring at the side of the DT-IPV vaccine is significantly higher than on the MMR vaccine side based on McNemar’s chi-squared test result: χ^2^ = 488.51, *p* < 0.001.

A comprehensive overview of all reported AEFIs is provided in Table S1. This includes reactions such as aphthous ulcers in the mouth, which were observed one week after vaccination in two children. One of these children had no prior history of such symptoms. Two children experienced nosebleeds: in one case, the nosebleeds began three days post-vaccination and recurred over the course of a week; in the other case, a single spontaneous nosebleed occurred two weeks after vaccination. One child developed a swollen, red, and painful arm, which was diagnosed by a general practitioner as an allergic reaction. No systemic symptoms were present, and no antihistamine treatment was administered.

There were four serious AEFIs reported after vaccination in the cohort; hospitalisation was required for these four children. One child experienced severe headache, nausea, and vomiting approximately nine hours post-vaccination, necessitating a one-day hospital stay. Treatment included antiemetic medication and intravenous fluids. It was reported that this child had a history of prolonged vomiting episodes. Recovery occurred within five days.

Another child developed symptoms of headache, vomiting, and confusion four weeks after vaccination. The child was hospitalised for four days and treated with unspecified medication.

Two children were hospitalised due to infections. One case involved appendicitis, with symptoms of headache, nausea, abdominal pain, and fever beginning within hours of vaccination. Hospitalisation and surgical removal of the appendix occurred two days later, with full recovery following the procedure. Another child was admitted to the hospital three weeks post-vaccination with low oxygen saturation due to pneumonia and was treated with antibiotics.

### 2.4. Consumption of Care

Parents or caregivers reported a range of actions taken in response to AEFIs occurring in children. More than one action per child could be reported. In most cases, the AEFI was discussed with a healthcare professional (65 times reported). Medication was administered in 15 cases, while 12 children were treated without medication. In another 12 instances, the AEFIs were both discussed and treated without medication, and in 6 cases, it was discussed and followed by medication administration. Additionally, 25 participants described other actions, including diagnostic procedures, dietary interventions or referrals to specialists. The cases in which a hospitalisation occurred have already been described above.

### 2.5. Prediction Model for Factors Contributing to Reported Adverse Events Following Immunisation (AEFIs)

**Figure 3:**
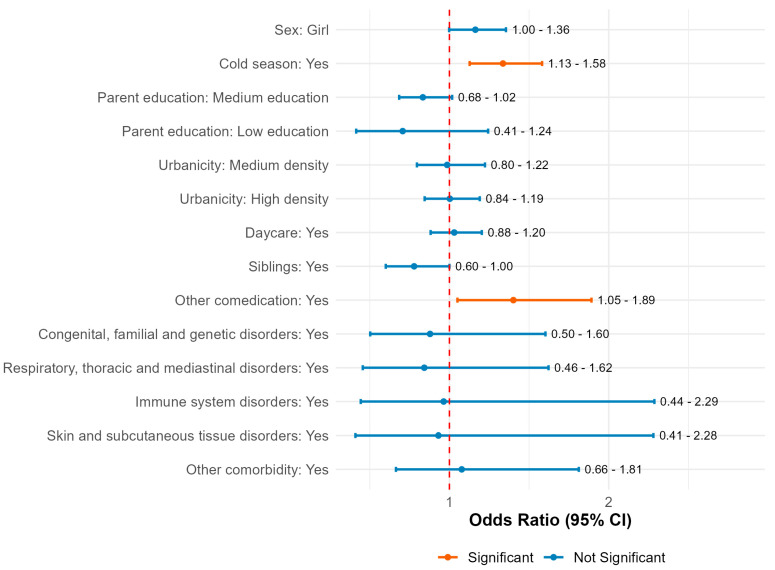
Results of the multivariable logistic regression analysis for factors contributing to the odds of reporting an AEFI.

Characteristics of the vaccinated children or their households, including education level of the parent who filled in the questionnaires (see Figure 3), were used in a logistic multivariable prediction model to determine whether there were factors which contributed to the reporting of an AEFI. The use of any analgesic medication could not be taken into account as a variable because we could not distinguish between medication given as prophylaxis or treatment. Small but significant effects can be seen for the vaccination being given in the cold season (1.34; 95% CI 1.13–1.58) and for other comedication being used (1.40; 95% CI 1.05–1.89).

### 2.6. Time to Onset, Duration, and Burden of AEFIs

**Figure 4:**
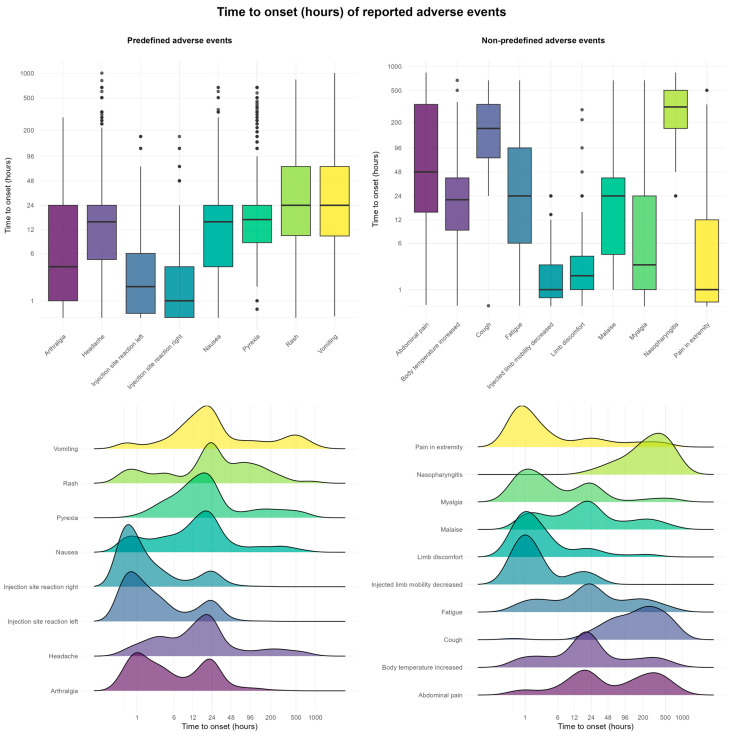
Combined box and ridgeline plots for time to onset of AEFI (both predefined and non-predefined).

Our findings indicate that most predefined AEFIs occur within 48 h following vaccination. Local reactions at or around the injection site—such as redness, pain, or swelling—typically develop shortly after vaccination. A portion of these AEFIs arise within the first hour, including pain experienced at the time of injection. Symptoms such as rash, vomiting, and fever were observed to occur several days post-vaccination in some children. In the ridgeline plots in Figure 4, a secondary peak is visible for certain AEFIs, which aligns with known patterns associated with the MMR vaccine.

**Figure 5:**
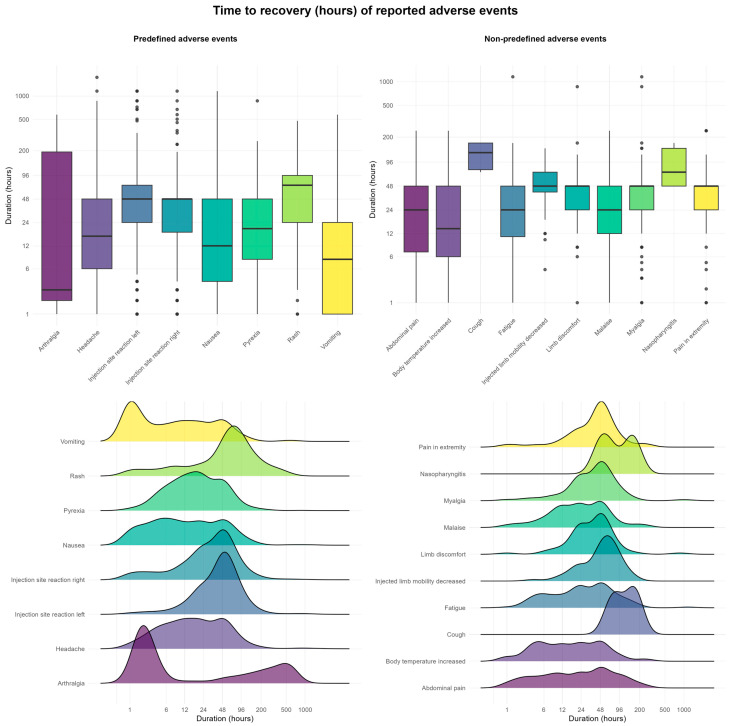
Combined box and ridgeline plots for duration of AEFI (both predefined and non-predefined).

The recovery time for AEFIs generally follows the expected pattern, with most AEFIs from the predefined checklist resolving within approximately three days (Figure 5). However, symptoms such as skin rash and joint pain may persist in a small subset of children for more than a week post-vaccination.

**Figure 6:**
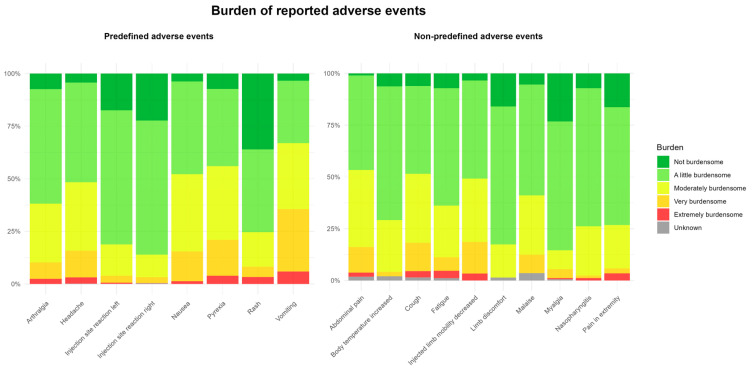
Graphical representation of the perceived burden of AEFIs (both predefined and non-predefined).

As shown in Figure 6, most of the commonly reported AEFIs were reported by parents as either not burdensome or only mildly burdensome for their child. Among the most frequently reported symptoms, vomiting was rated as the most burdensome, while injection site reactions were perceived as the least burdensome. 

## 3. Discussion

By following a defined group of vaccinated individuals over time, cohort event monitoring (CEM) studies can provide additional safety data to passive reporting systems. V-Safe in the US is an example of such a monitoring system, which has provided a wealth of information on vaccine safety in daily practice [12]. Having such monitoring systems in place enhances the ability to assess the frequency, timing, and severity of adverse events. CEM provides concrete data that can be used to explain what AEFIs to expect, when they might occur, and how long they typically last. This helps set realistic expectations for children and their parents or caretakers who follow the NIP and helps health professionals in their communication regarding AEFIs. By performing this study, we are able to provide updated information on the study performed fifteen years ago [11].

In this monitoring study, 9-year-old children were followed for 30 days after receiving the MMR-DT-IPV vaccination to assess the pattern, timing, and burden of AEFIs. In total, data was available for 3590 children based on at least one completed questionnaire. Of these, 73.1% reported at least one AEFI based on questionnaires their parents or guardians filled in. Commonly known AEFIs such as injection site reactions and headache, were frequently observed in this study and serious AEFIs were rare, in accordance with previous studies [11,13]. The latency and recovery times showed that reactions often occurred relatively soon after vaccination and were often self-limiting. The delayed onset of some systemic reactions is likely attributable to the MMR vaccine, since this is a live attenuated vaccine [14,15]. However, a previous study from Lareb on data on MMR AEFIs from the spontaneous reporting system found that the first MMR vaccine gave a bimodal pattern of latency periods for AEFIs with a clear second peak between 5 and 12 days. Meanwhile, the second MMR vaccine given at 9 years of age only showed a peak in the first few days following vaccination. The second peak of AEFI reporting was almost absent. These results suggest that a second MMR vaccination is less reactogenic than the first MMR vaccine [16]. A plausible explanation for this phenomenon is that the viremia induced by the second vaccination is effectively suppressed by the immune response elicited by the first vaccination, resulting in fewer adverse reactions. Although age may also contribute to the reduced reactogenicity, pre-existing immunity due to prior measles vaccination is considered a more likely mechanism [16,17,18].

Most children did not perceive the AEFI as highly burdensome. Vomiting was reported as the most burdensome AEFI. A rash resembling a mild form of measles is a known adverse reaction of the MMR vaccine [19]. A rash following MMR-DT-IPV vaccination was generally perceived as less burdensome by participants, possibly due to prior information provided about this AEFI or because the MMR vaccine was administered once before at a younger age. As previously mentioned, studies have found a second MMR vaccination to be less reactogenic [16,17,18].

In a multivariable logistic regression analysis, we studied the contribution of factors relating to the child, like their sex and comorbidities, vaccination date, and factors relating to the household they grow up in. We found a small but significant effect for vaccination being given in the cold season vs. warmer months in the reporting of AEFIs. However, when we further stratified the dataset to injection site reactions only, this factor was no longer significant. In this study we collected AEFIs defined as ‘*an adverse event following immunisation is any untoward medical occurrence which follows immunisation and which does not necessarily have a causal relationship*’. An experienced AEFI can also be due to nocebo effects or background incidence [20,21]. Our hypothesis is that in the cold season in The Netherlands, there is a higher chance of viral infections like rhinovirus or norovirus infection, for which symptoms might be attributed to the vaccination. The correlation between “urbanicity” and “participation in after-school care” may further affect the risk of infection, which could be a potential confounding factor.

In addition, the use of concomitant medication was significantly associated with the reporting of AEFIs. However, none of the larger groups of comorbidities in our cohort were significantly associated with higher odds of reporting an AEFI. Since the medications used by the children were very diverse, the clinical relevance of this finding is limited at the moment. The higher AEFI rate in the concomitant medication group may be attributed to the underlying diseases of the children themselves, rather than the interaction between medications and vaccines.

For female sex, the point estimate 1.16 (95% CI 1.00–1.36) suggests a small positive association, however there is insufficient evidence to conclusively rule out no effect. Previous studies have found higher reactogenicity relating to female sex [22,23,24]. For the MMR vaccine, a self-controlled case series study observed a significant effect of female sex on the relative incidence (1.08; 95% CI 1.03 to 1.14) of serious AEFIs after MMR vaccination at 12 months. This effect was not significant for MMR vaccination given in younger age groups [25]. Differences in vaccine response are not solely attributable to sex hormones but are also influenced by genetic factors [26].

This study is part of a large CEM study for NIP vaccines in The Netherlands. Children who were part of this study on the MMR-DT-IPV vaccination and followed the NIP were also invited a year later to participate in questionnaires on the Human papillomavirus infection (HPV) vaccinations, given around 10 years of age. Results of the CEM study for HPV vaccines have been reported separately [22].

### 3.1. Study Strengths and Limitations

All questionnaires were completed by parents or guardians of vaccinated children. Consequently, the reported burden of AEFIs may reflect the proxy’s perception rather than the child’s actual experience, potentially leading to over or underestimation of severity. Additionally, although guidelines specify which vaccine should be given in which arm, parents may mistakenly report injection site reactions based on their own left/right orientation rather than that of the child. Despite this limitation, patient (via proxy)-reported outcomes are of added value, especially for AEFIs that are less serious or not registered in, for example, a clinical setting. Cohort event monitoring studies provide deeper insights into the nature of reported AEFIs. Unlike spontaneous reporting systems, they enable the calculation of incidence rates, offering a clearer picture of vaccine safety. These studies help answer the very questions parents often ask: What kinds of AEFIs are likely to occur after vaccination? How long do they last? And what is their expected burden?

While patient-reported outcomes (PROs) may not align precisely with clinical diagnostic criteria and are subject to individual variability in symptom perception and reporting behaviour, they offer valuable insights into the real-world experiences of vaccine recipients. A key advantage of PROs is their ability to capture common AEFIs—such as local and systemic reactions—that are readily recognised by vaccine recipients but may not prompt medical consultation. As such, these events are often only reliably reported by the individuals themselves or, in the case of children, by parents or caregivers. Although the inclusion of medical records for verification of AEFIs could be a consideration, such data are frequently unavailable and are likely to underrepresent medically non-serious events. Therefore, PROs remain an essential source of information for capturing the full spectrum of vaccine-related experiences in the population [27].

The participant group in this study is likely not fully representative of all children enrolled in the NIP. This is partly due to the inclusion criterion requiring parents/guardians to have sufficient proficiency in Dutch and have access to a digital device with internet connectivity. This may have caused hurdles for low-income households and for those less fluent in Dutch such as people belonging to the Dutch immigrant population, which was estimated as approximately 16 percent of people residing in The Netherlands [28]. Additionally, the educational level of participating parents/guardians was generally higher than the national average in The Netherlands [29]. Approximately 6.8% of the children for whom questionnaires were completed had a medical condition. This is lower than estimates from previous research among Dutch children, which suggest that approximately 1 in 5 children have a chronic condition [30]. This might lead to some bias in our estimates due to the healthy user effect, or in this case, the healthy vaccinee effect [31]. The AEFI reporting patterns of children in likely under-represented groups may differ significantly from those in households with high parental education levels, e.g., these households may have a lower awareness of AEFIs, leading to potentially lower reporting rates.

Participation and dropout during the follow-up period may have been selective. It is expected that participants were more likely to complete questionnaires if any health events actually occurred. It is also possible that some participants with missing questionnaires no longer followed the NIP.

Lastly, in this study, we investigated adverse events following immunisation (AEFIs), defined as any untoward medical occurrence that follows immunisation, which does not necessarily have a causal relationship with the vaccine. An AEFI may manifest as an unfavourable or unintended sign, abnormal laboratory finding, symptom, or disease. According to the World Health Organization (WHO) manual on causality assessment of AEFIs [32], such assessments should be conducted at multiple levels. At the population level, the objective is to determine whether a causal association exists between a specific vaccine and a particular AEFI. At the individual case level, the assessment involves reviewing existing evidence and applying logical reasoning to evaluate whether a specific AEFI in an individual is causally linked to the administered vaccine. The primary aim of an individual-level causality assessment is to address the question: “Did the vaccine administered to this individual cause the reported event?” While definitive conclusions are rarely possible—except in cases of well-established reactions such as injection site pain—the process involves systematically evaluating all potential causes to determine whether the evidence supports, refutes, or is inconclusive regarding a causal relationship with the vaccine. In our analysis, we focused on AEFIs at the population level within our cohort. Individual-level causality assessments were not conducted for all reported AEFIs, except in cases classified as serious or involving unexpected (unlabelled) events. Consequently, some AEFIs observed in our cohort may represent coincidental occurrences rather than true adverse reactions.

### 3.2. Future Studies

Since 1 January 2025, the NIP schedule has been updated. MMR vaccination has been rescheduled to approximately 3 years of age and DT-IPV vaccination will be postponed to age 14. Our study results will serve as a baseline to conduct further comparisons of the AEFI profile of MMR and DT-IPV vaccination given at these new ages. In addition, more work is needed to ensure the capture of a selection of participants that is representative of the entire Dutch population.

## 4. Materials and Methods

This study was conducted as part of a Lareb vaccine monitoring project [33], employing a longitudinal cohort design. Parents or guardians of participating 9-year-olds completed questionnaires following vaccination to report the presence or absence of an AEFI.

**Figure 7:**
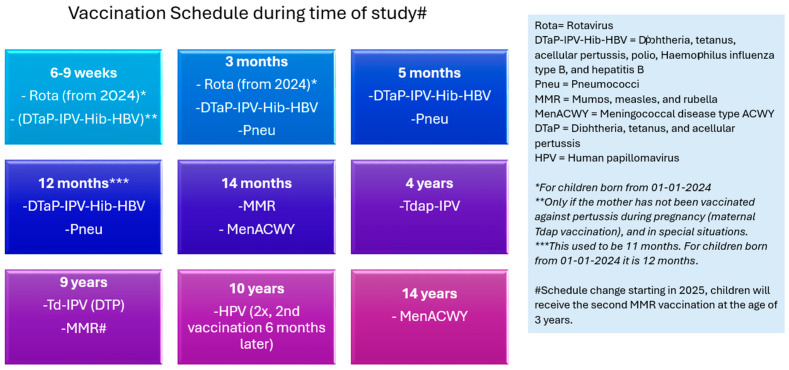
Graphical representation of vaccinations and vaccination moments in the Dutch NIP at the time of this study [34].

Eligibility criteria included the following: parents/guardians aged 16 or older, children born in 2013, 2014, or 2015, and children scheduled to receive vaccinations per the NIP. Children with missing or non-standard birth years were excluded from analysis. Participants were required to be proficient in Dutch and have access to a digital device with internet connectivity. Only residents of The Netherlands were eligible. An additional exclusion criterion was an excessive delay between completion of the intake questionnaire and the vaccination date. The study period spanned from 1 April 2022 to 29 July 2024. Figure 7 presents the Dutch NIP at the time of the study.

### 4.1. Registration and Intake Questionnaire

From August 2022 to February 2024, parents/guardians were invited via a letter to participate, using the infrastructure of the RIVM’s Department of Vaccine Supply and Prevention Programmes (DVP). The invitation included a flyer with details of a dedicated website where the privacy policy, project proposal, and FAQs were listed. During registration, participants received detailed study information and completed an informed consent form. Upon confirmation, they received a baseline questionnaire and digital questionnaires addressing post-vaccination symptoms.

### 4.2. Questionnaire Timing

For the DT-IPV vaccine (REVAXIS^®^), most AEFIs are expected within 48 h and typically resolve within 1–2 days [14]. Therefore, the first questionnaire was administered one week post-vaccination. A follow-up questionnaire was sent two weeks later. Since MMR (M-M-RVAXPRO^®^) may cause AEFIs up to three weeks post-vaccination [15], the follow-up period was extended. A final questionnaire was sent on day 30, by which time most symptoms are expected to have resolved. Reminders were issued 2 and 5 days after each questionnaire if not yet completed. Participants could voluntarily withdraw from the study at any time.

### 4.3. Questionnaire Coding

AEFIs and comorbidities were coded with the Medical Dictionary for Regulatory Activities (MedDRA^®^) [35]. Vaccines and drugs were coded with the Dutch drug database (G-Standaard) [36].

### 4.4. Data Analysis

The questionnaires collected demographic and health-related data, including sex, age, comorbidities, number of children in the household, parental or guardian education level, postal code of residence, participation in after-school care, receipt of non-NIP vaccinations, vaccination date, AEFIs, and care-consumption after AEFI.

AEFIs were categorised into common, expected adverse events (e.g., injection site reactions, headache, fever, nausea, vomiting, joint pain, and rash) selectable from a predefined list, and less common symptoms, which could be entered in a free-text field. For fever the diagnostic criterium was a body temperature measured as 38 °C or higher. Body temperatures that were lower or unmeasured were coded as body temperature increased instead of pyrexia. The medical seriousness of AEFIs was assessed using international criteria [37], including whether hospitalisation was required. Use of (antipyretic) medication around the time of vaccination, either prophylactic or therapeutic, was also recorded.

Time to onset (TTO) and duration (in hours) of AEFIs were documented. The perceived burden of AEFI was rated on a 5-point scale from “not burdensome” to “very burdensome.” Descriptive statistics are presented in summary tables and figures per vaccination moment. Because two vaccinations are given simultaneously, it is not possible to assign systemic reactions to one of the vaccines. For injection-site reactions, participants listed the arm(s) the reaction occurred in. These could then be assigned to the vaccine that was given. According to national guidelines the MMR vaccine (M-M-RVAXPRO^®^) is given in the right arm and DT-IPV vaccine (REVAXIS^®^) in the left arm [38]. Differences between frequencies of local reactions at the DT-IPV and MMR injection sites were tested using the McNemar test for paired proportions. Significance (2-sided) was based on *p* < 0.05. For the graphical representation of the TTO and TTR, both boxplots and ridgeline plots were used.

We performed multivariable logistic regression for the outcome ‘any AEFI’. Included variables were the child’s sex, existence of comorbidity (subgroups based on MedDRA System Organ Classes: Congenital, familial, and genetic disorders; Respiratory, thoracic, and mediastinal disorders; Immune system disorders; Skin and subcutaneous tissue disorders; and Other comorbidity), use of concomitant medication given for any comorbidity, having at least one sibling in the household, participation in after-school care (as a binary variable) and season in which the vaccination took place (either ‘colder season’ from October to March or ‘warmer season’ from April to September), parent educational level (categorised as low, medium, or high [39]), and urbanicity of the living environment. The level of urbanicity was based on calculations by Lareb using non-public microdata on postal codes, urbanisation category, and environmental address density from Statistics Netherlands [40]. We tested for collinearity between variables [41]. Data analysis was performed in R, version 4.5.1.

## 5. Conclusions

AEFIs very frequently occurred after MMR and DT-IPV vaccination, given simultaneously to children at 9 years of age in this Dutch CEM study. The great majority of reported reactions were non-serious and self-limiting and consistent with those listed in the official product information for the MMR and DT-IPV vaccines. This study provides further insight into the timing and duration of AEFIs after MMR and DT-IPV vaccination. AEFIs were mostly perceived as a little or moderately burdensome. This study helps with providing detailed insight into the adverse event profile of these vaccines, which helps to set realistic expectations for children and their parents or caretakers who follow the NIP and helps health professionals in their communication regarding on AEFIs.

## Figures and Tables

**Table 1:** Characteristics of participants after baseline and Questionnaire 1.

Characteristics of Participants (N = 3590 Questionnaire 1)	
**Questionnaires filled in by unique participants, n (%)**	
Baseline questionnaire	3590 (100)
First questionnaire (7 days after vaccination)	3274 (91.2)
Follow-up questionnaire (14 days after vaccination)	2636 (73.4)
**Sex, n (%)**	
Boy	1808 (50.4)
Girl	1780 (49.6)
Missing	2 (0.1)
**Birth cohort, n (%)**	
2013	756 (21.1)
2014	2730 (76.0)
2015	104 (2.9)
**Existing medical conditions (MedDRA System Organ Class level), n (%)**	
Congenital, familial, and genetic disorders	60 (1.7)
Respiratory, thoracic, and mediastinal disorders	58 (1.6)
Immune system disorders	42 (1.2)
Skin and subcutaneous disorders	36 (1.0)
Psychiatric disorders	20 (0.6)
Nervous system disorders	18 (0.5)
Gastrointestinal disorders	16 (0.4)
Cardiac disorders	11 (0.3)
Eye disorders	6 (0.2)
Metabolism and nutrition disorders	6 (0.2)
Musculoskeletal and connective tissue disorders	5 (0.1)
Renal and urinary disorders	5 (0.1)
Ear and labyrinth disorders	4 (0.1)
Blood and lymphatic system disorders	3 (0.1)
Endocrine disorders	2 (0.1)
Vascular disorders	1 (0.0)
General disorders and administration site conditions	1 (0.0)
Infections and infestations	1 (0.0)
Investigations	1 (0.0)
Neoplasms—benign, malignant, and unspecified (incl. cysts and polyps)	1 (0.0)
Surgical and medical procedures	1 (0.0)

**Table 2:** AEFIs reported after MMR and DT-IPV vaccination (n, %).

Reported AEFIs		Total Participants N = 3590
	At least one AEFI	2625 (73.1)
	At least one predefined AEFI	2322 (64.7)
	At least one other AEFI	969 (27.0)
	At least one serious AEFI	4 (0.1)
**Predefined AEFIs (MedDRA PT)**		
	Injection site reaction (grouped per child)	1872 (52.1)
	Headache	646 (18.0)
	Pyrexia	495 (13.8)
	Nausea	293 (8.2)
	Arthralgia	244 (6.8)
	Vomiting	117 (3.3)
	Rash	60 (1.7)
**Top 10 Non-Predefined AEFIs (MedDRA PT)**		
	Fatigue	166 (4.6)
	Myalgia	162 (4.5)
	Abdominal pain	103 (2.9)
	Pain in limb	85 (2.4)
	Naso-pharyngitis	84 (2.3)
	Limb discomfort	68 (1.9)
	Cough	66 (1.8)
	Limb mobility decreased	58 (1.6)
	Malaise	56 (1.5)
	Body temperature increased	48 (1.3)

**Table 3:** AEFIs reported after MMR and DT-IPV vaccination (n, %).

Injection Site Reactions: MMR (Right Side) and DT-IPV (Left Side) (N = 3590) (n, %)	
Left	1585 (44.2) *
Right	843 (23.5) *
Unknown	120 (3.3)

* Statistically significant difference based on McNemar’s chi-squared test result: χ^2^ = 488.51, *p* < 0.001.

## Data Availability

The data from the CEM study cannot be made fully publicly available due to the General Data Protection Regulation (GDPR) and the general privacy regulation of the Pharmacovigilance Centre Lareb. Request to access the data may be granted on reasonable request via the corresponding author.

## Supplementary Materials

The following supporting information can be downloaded at https://www.mdpi.com/article/10.3390/ph18111635/s1, Table S1. List of all reported AEFIs in the study.

## Abbreviations

The following abbreviations are used in this manuscript:

AEFI	Adverse event following immunisation
CEM	Cohort event monitoring
CIOMS	Council for International Organizations of Medical Sciences
DT-IPV	Diphtheria, pertussis, and inactivated poliomyelitis
Lareb	The Dutch Pharmacovigilance Centre
MedDRA	Medical Dictionary for Regulatory Activities
MMR	Measles, mumps, and rubella
NIP	National Immunisation Programme
PRO	Patient-reported outcome
RIVM	National Institute for Public Health and the Environment
TTO	Time to onset
TTR	Time to recovery
