# Supplementary material for: Longitudinal Cohort Event Monitoring of MMR and DT-IPV Vaccination at 9 Years of Age in The Netherlands
Source: Pharmaceuticals (Basel). 2025 Oct 29;18(11):1635. doi: 10.3390/ph18111635 (PMC12655693; doi:10.3390/ph18111635)
Supplement: Supplementary file 1 [file pharmaceuticals-18-01635-s001.zip › pharmaceuticals-3916750-supplementary.pdf]

pharmaceuticals

MDPI

Article

Longitudinal Cohort Event Monitoring of MMR and DT-IPV Vaccination at 9 Years of Age in The Netherlands

Monika Raethke[1], Jeroen Gorter[1], Rachel Kalf[1], Leontine van Balveren[1], Sanne Boetzkes[1], Rana Jajou[1] and Florence van Hunsel[1,2]*

[1] Netherlands Pharmacovigilance Centre Lareb, Goudsbloemvallei 7, 5237 MH 's-Hertogenbosch, The Netherlands;
[2] Department of PharmacoTherapy—Epidemiology & Economics, Groningen Research Institute of Pharmacy (GRIP), University of Groningen, Broerstraat 5, 9712 CP Groningen, The Netherlands
* Correspondence: f.vanhunsel@lareb.nl

Table S1. All reported AEFI.

**AEFI (MedDRA term PT level)**	**N**	**%**
Injection site reaction	1893	85,7
Headache	674	30,5
Pyrexia	511	23,1
Nausea	301	13,6
Arthralgia	244	11
Fatigue	170	7,7
Myalgia	163	7,4
Vomiting	120	5,4
Abdominal pain	105	4,8
Pain in extremity	85	3,8
Nasopharyngitis	84	3,8
Limb discomfort	68	3,1
Cough	67	3
Rash	61	2,8
Injected limb mobility decreased	58	2,6
Malaise	56	2,5
Body temperature increased	48	2,2
Listless	42	1,9
Oropharyngeal pain	41	1,9
Dizziness	36	1,6
Syncope	25	1,1
Diarrhoea	21	1
Extensive swelling of vaccinated limb	21	1
Influenza like illness	19	0,9
Pallor	15	0,7
Decreased appetite	13	0,6
Ear pain	8	0,4

Musculoskeletal stiffness	8	0,4
Insomnia	7	0,3
Rash pruritic	7	0,3
Lymphadenopathy	6	0,3
Axillary pain	5	0,2
Erythema	5	0,2
Impetigo	5	0,2
Asthenia	4	0,2
Crying	4	0,2
Ear inflammation	4	0,2
Eczema	4	0,2
Influenza	4	0,2
Pneumonia	4	0,2
Pruritus	4	0,2
Chest pain	3	0,1
Gastroenteritis viral	3	0,1
Irritability	3	0,1
Muscular weakness	3	0,1
Nasal congestion	3	0,1
Peripheral swelling	3	0,1
Vaccination error	3	0,1
Visual impairment	3	0,1
Aphthous ulcer	2	0,1
Body temperature abnormal	2	0,1
Body temperature normal	2	0,1
COVID-19	2	0,1
Chills	2	0,1
Condition aggravated	2	0,1
Delirium febrile	2	0,1
Dysphonia	2	0,1
Epistaxis	2	0,1
Eye inflammation	2	0,1
Feeling cold	2	0,1
Haematoma	2	0,1
Hyperhydrosis	2	0,1
Infection susceptibility increased	2	0,1
Lymph node pain	2	0,1
Migraine	2	0,1
Muscle spasms	2	0,1
Neck pain	2	0,1
Otorrhoea	2	0,1
Pain	2	0,1

Palpitations	2	0,1
Papule	2	0,1
Paraesthesia	2	0,1
Productive cough	2	0,1
Rash erythematous	2	0,1
Rhinorrhoea	2	0,1
Somnolence	2	0,1
Viral infection	2	0,1
Abnormal behaviour	1	0
Anal eczema	1	0
Appendicitis	1	0
Asthma	1	0
Atypical pneumonia	1	0
Autism spectrum disorder	1	0
Axillary mass	1	0
Back pain	1	0
Body tinea	1	0
C-reactive protein increased	1	0
Compulsions	1	0
Confusional state	1	0
Constipation	1	0
Cyanosis	1	0
Cystitis	1	0
Depressed mood	1	0
Discomfort	1	0
Dizziness postural	1	0
Dry mouth	1	0
Dysgeusia	1	0
Dyskinesia	1	0
Dyspnoea	1	0
Ear swelling	1	0
Emotional disorder	1	0
Erythema annulare	1	0
Erythema induratum	1	0
Erythema infectiosum	1	0
Eye irritation	1	0
Eye pain	1	0
Eye pruritus	1	0
Feeling of body temperature change	1	0
Foreign body in throat	1	0
Frequent bowel movements	1	0
Furuncle	1	0

Gastrointestinal pain	1	0
Groin pain	1	0
Heart rate increased	1	0
Hordeolum	1	0
Hypersensitivity	1	0
Hypotension	1	0
Increased appetite	1	0
Irritable bowel syndrome	1	0
Lip erythema	1	0
Liver tenderness	1	0
Mood altered	1	0
Mood swings	1	0
Muscle discomfort	1	0
Musculoskeletal discomfort	1	0
Nightmare	1	0
Oral discomfort	1	0
Oral herpes	1	0
Oxygen saturation decreased	1	0
Pharyngitis	1	0
Photosensitivity reaction	1	0
Pulmonary pain	1	0
Rash papular	1	0
SARS-CoV-2 test negative	1	0
Sever's disease	1	0
Sinusitis	1	0
Sneezing	1	0
Swelling face	1	0
Taste disorder	1	0
Tic	1	0
Tongue discolouration	1	0
Tongue erythema	1	0
Tonsillar hypertrophy	1	0
Tremor	1	0
Urticaria	1	0

Disclaimer/Publisher's Note: The statements, opinions and data contained in all publications are solely those of the individual author(s) and contributor(s) and not of MDPI and/or the editor(s). MDPI and/or the editor(s) disclaim responsibility for any injury to people or property resulting from any ideas, methods, instructions or products referred to in the content.
